# Effects of different early cardiac rehabilitation exercise treatments on the prognosis of acute myocardial infarction patients receiving percutaneous coronary intervention

**DOI:** 10.1016/j.clinsp.2024.100408

**Published:** 2024-06-13

**Authors:** Huiying Liang, Xinhua Hu, Hongying Liao

**Affiliations:** a Department of Cardiovascular Medicine, Ganzhou People's Hospital, Ganzhou, China; b Department of Nursing, Shangyou County People's Hospital, Ganzhou, China

**Keywords:** Acute myocardial infarction, Cardiac rehabilitation, Cardio-pulmonary function, Exercise, Percutaneous coronary intervention

## Abstract

**Objectives:**

Exercise rehabilitation is the core of Cardiac Rehabilitation (CR) and will improve the prognosis of patients receiving Percutaneous Coronary Intervention (PCI surgery). The current study retrospectively analyzed the effects of different exercise-based CR strategies on the prognosis of AMI patients receiving PCI treatment.

**Methods:**

Clinicopathological information from 127 patients was collected and divided into different groups based on the exercise-based CR received, including Continuous Resistance Exercise (COR), Continuous Aerobic Exercise (COA), Interval Resistance Exercise (IVR), Interval Aerobic Exercise (IVA), Inspiratory Muscle Exercises (ITM), and Control. The differences regarding cardio-pulmonary function, hemodynamics, and life quality were analyzed against different CR strategies.

**Results:**

All the exercise-based CR strategies showed improving effects compared with patients in the Control group regarding cardio-pulmonary parameters, with IVR showing the strongest improving effects (IVR > ITM > COR > IVA > COA) (p < 0.05) at the first recoding point. However, the improving effects of exercise-based CR declined with time. Regarding the effects on hemodynamics parameters, the improving effects of exercise-based CR were only observed regarding LVEF, and the effects of IVR were also the strongest (IVR > COR > ITM > COA > IVA) (p < 0.05). Similar improving effects were also observed for 6MWT and life quality (IVR showing the strongest improving effects) (p < 0.05), which all declined three months after the surgery.

**Conclusions:**

The current study showed that exercise-based CRs had better improving effects than the normal nursing strategy on the prognosis of AMI patients receiving PCI surgery.

## Highlights

• Exercise-based CR improved cardio-pulmonary function of patients receiving PCI.
• Exercise-based CR improved hemodynamics parameters of patients receiving PCI.
• IVR exercise showed much better effects than other strategies.

## Introduction

Acute Myocardial Infarction (AMI) is a severe form of Acute Coronary Syndrome (ACS), which is caused by sudden obstruction of blood flow to the vein.^1^^,^^2^ Based on clinical symptoms, AMI can be divided into three types, including unstable angina, non-st-elevation Myocardial Infarction (NSTEMI), and ST-Elevation Myocardial infarction (STEM). The average incidence of AMI is ca. 2 % to 5 %, ranking the highest of all cardiovascular diseases and accounting for over 30 % of all deaths. In China, the mortality due to AMI keeps increasing gradually,^3^ and the number of coronary events is expected to increase by 69 % and the number of deaths will increase by 64 % between 2020 and 2029,^4^ which has become a great burden to the public health system and caregivers. Currently, the most widely employed treatment strategy for AMI in clinics is Percutaneous Coronary Intervention (PCI). The technique has the advantages such as restoring cardiac blood perfusion, ameliorating clinical symptoms, and preventing disease progression, and thus will reduce short-term mortality.^5^ However, Adverse Cardiovascular Events (ACE) still occur in some AMI patients after PCI. For instance, the incidence of cardiovascular end-point events is about 5 % to 15 %,^6^, ^7^, ^8^ and the recurrent rate of chest pain is as high as 50 %.^9^ These adverse events lead to an increase in the amount spent on healthcare due to growing re-hospitalization. Thus, the effective improvement of the prognosis of AMI patients receiving PCI has become a critical issue in a clinic.

Cardiac Rehabilitation (CR) arises at a historic moment with the implementation of comprehensive management on CVD patients, and has shown promising beneficial effects on patients after Acute Coronary Syndromes (ACSs), impaired Left Ventricular Ejection Fraction (LVEF), and other clinical presentations of coronary atherosclerosis (CAD).^10^, ^11^, ^12^]. Exercise rehabilitation is the core of cardiac rehabilitation,^13^ and previous analysis shows that exercise-based CR improves angina pectoris, myocardial infarction, and restenosis in CHD patients.^14^^,^^15^ Additionally, it is inferred that the earlier the exercise starts, the better the outcome the CR achieves. There are few research or case reports on patients’ exercise rehabilitation three months after coronary revascularization. Regarding the effects on AMI patients treated with PCI, numerous studies have indicated the efficacy and safety of the prognosis after receiving exercise-based CR strategies.^16^^,^^17^ For instance, the study by Zhuo et al. shows that a 10-day-period CR procedure for patients with ST-segment elevation acute myocardial infarction after PCI substantially improved the cardiac function and psychological state of the patients.^18^ However, the application of exercise-based CR strategies to AMI patients receiving PCI lacks standard procedures, and the difference regarding the efficacy of different exercises is yet to be assessed. The comparison between different exercises during CR will provide valuable information for improving the practice of exercise-based CR strategies on AMI patients receiving PCI. Thus, in the current study, the authors performed a retrospective analysis of the effects of different exercise-based CR strategies on the prognosis of 127 AMI patients receiving PCI from Jan 2022 to May 2023 in the studied hospital. The parameters regarding cardiac function and prognosis achieved in the present study's hospital were retrieved and analyzed to assess the improving exercise types.

## Methods

### Patients

The current analysis included 127 AMI patients receiving PCI treatment in the present study's hospital from Jan 2022 to May 2023. All the included cases met the inclusion criteria covered the following: stable angina pectoris (> half an hour) with episodic post sternal dull pain accompanied by sweating, nausea, dyspnea, suffocation, and even syncope and other clinical symptoms; the levels of serum biomarkers of myocardial injury exceeds the upper limit of the reference value for at least once; history of elective PCI (< 3 months) and complete coronary revascularization; LVEF > 45 % (assessed after complete coronary revascularization before discharge from hospital or later, before beginning of the rehabilitation program); consent to be included in the rehabilitation program; the patient's condition allowing the treadmill rehabilitation program to be conducted according to a specific protocol, exercise load on treadmill test before rehabilitation program.

Clinicopathological information retrieved from cases with the presence of contraindications to rehabilitation training, other disease states making it impossible to conduct the rehabilitation program, and life-threatening cardiac arrhythmias were not used in the analysis. The study was approved by the ethics committee of Ganzhou People's Hospital for the related screening, inspection, and data collection (approval nº 2022A125) and was performed in accordance with the Declaration of Helsinki and followed STROBE Statement. All the patients had signed a written informed consent form.

### Rehabilitation program

The cases in the current study were subjected to different types of exercise-based CR including A) Continuous Resistance exercises (COR) such as sit-ups and squats; B) Continuous Aerobic exercise (COA) such as swim and jogging; C) Interval Resistance exercises (IVR) such as sit-up and squat with the interval between COR; D) Interval Aerobic exercise (IVA) such as HIIT; E) Inspiratory Muscle exercises (ITM) such as deep breathing exercises and intercostal respiration exercises; F) Cases underwent regular nursing after the surgery were employed as Control group in the current analysis, and could have regular some regular exercises as they wished.

### Data collection

All the included patients were admitted to the Cardiac Rehabilitation clinic of this hospital for cardiopulmonary exercise tests one and three months after discharge: briefly, patients were subjected to a treadmill scheme test using the Exercise ECG Exercise Plate Tester (AT-104 PC, Swiss Schiller, SCHILLER).

The tests should be terminated immediately if any of the following occurred, and emergency management was given according to the patient's condition: A) Reaching the target heart rate; B) The appearance of typical angina; C) The presence of obvious symptoms of dyspnea, pallor, cyanosis, dizziness, dizziness, unsteady gait, movement disorders, ischemic claudication; D) Discomfort or pain in the lower limbs that increases with exercise; E) Presence of ST segment flat or downward slope descending ≥0.25 mV or injured ST elevation ≥ 2.0 mV; F) occurrence of arrhythmias G) Systolic blood pressure does not rise or decrease > 20 mmHg during exercise; hypertension, systolic blood pressure > 220 mmHg; H) Exercise-induced intraventricular block; I) The patient asks for the end motion.

The data of two tests, including heart rate at anaerobic threshold, peak heart rate, oxygen uptake at anaerobic threshold, peak oxygen uptake, metabolic equivalent at anaerobic threshold, peak metabolic equivalent, maximum oxygen pulse, anaerobic threshold RER, peak RER, Carbon Dioxide Ventilation Equivalent slope (VE/VCO_2_ slope), ΔVO_2_/ΔWR, Six-Minute Walk Test (6MWT) etc., were collected for the subsequent analyses and assessments.

Hemodynamic parameters including end-diastolic anteroposterior diameter and Left Ventricular Ejection Fraction (LVEF) were also collected from records at the time of admission and three months after discharge.

### Statistical analysis

Continued data were expressed as mean ± Standard Deviation (SD). The differences in continuous data were analyzed using ANOVA followed by post-hoc Tukey test. Difference between two groups was analyzed using Student's *t*-test for normal distribution data or Mann-Whitney *U* test for abnormal distribution data. Categoric data were expressed as numbers or proportions, and the difference was analyzed with the Chi-Square test. Significance was accepted when the two-tailed p-value was smaller than 0.05. All the statistical analyses and graph plotting were conducted using GraphPad Prism version 8.0.0 for Windows (GraphPad Software, San Diego, California USA, www.graphpad.com).

## Results

### Patients’ characteristics

The current analysis respectively included 127 AMI patients receiving PCI treatment, including 89 males and 38 females. Based on the exercise-based CR types following during the recovery phase, the patients were divided six groups. For Control group, 21 patients (average age 53.08 ± 11.80 years old), including 14 males and 7 females were enrolled; for COR group, 18 patients (average age 54.61 ± 9.37 years-old), including 11 males and 7 females were enrolled; for COA group, 19 patients (average age 58.11 ± 12.81 years-old), including 13 males and 6 females were enrolled; for IVA group, 21 patients (average age 55.42 ± 12.54 years-old), including 13 males and 8 females were enrolled; for IVR group, 22 patients (average age 53.61 ± 10.22 years-old), including 16 males and 6 females were enrolled; for ITM, 26 patients, including 14 males (average age 55.07 ± 13.67 years-old) and 12 females were included. As shown in Table 1, there was no significant difference regarding the parameters such as BMI, age, male proportion, hypertension proportion, and diabetes proportion upon admission to the present study's hospital (Table 1).

**Table 1:** Clinicopathological information.

	Control (n = 21)	COR (n = 18)	COA (n = 19)	IVA (n = 21)	IVR (n = 22)	ITM (n = 26)	p-value
**Age (year)**	53.08 ± 11.80	54.61 ± 9.37	58.11 ± 12.81	55.42 ± 12.54	53.61 ± 10.22	55.07 ± 13.67	0.575
**BMI (kg/m^2^)**	25.07 ± 3.27	24.03 ± 2.53	25.18 ± 2.89	22.15 ± 3.32	25.41 ± 2.75	24.11 ± 3.12	0.689
**Male, n (%)**	14 (70 %)	11 (61.1 %)	13 (68.4 %)	13 (61.9 %)	16 (72.7 %)	14 (53.8 %)	0.132
**Hypertension, n (%)**	6 (28.5 %)	3 (16.7 %)	5 (26.3 %)	5 (23.8 %)	7 (31.8 %)	4 (15.3 %)	0.082
**Diabetes, n (%)**	3 (14.2 %)	2 (11.1 %)	2 (10.5 %)	3 (14.2 %)	4 (18.1 %)	6 (23.1 %)	0.107

### Effects of different exercise-based CR strategies on the cardio-pulmonary function of AMI patients receiving PCI

The data regarding the cardio-pulmonary function of AMI patients receiving PCI were collected and analyzed against different exercise-based CR strategies. For parameters including oxygen uptake at anaerobic threshold, peak oxygen uptake, metabolic equivalent at anaerobic threshold, peak metabolic equivalent, and maximum oxygen pulse, all the exercise-based CR strategies showed improving effects compared with patients receiving normal nursing (Table 2) (p < 0.05) at the first recording point (one month after the discharge). However, the improving effects were not observed for parameters including heart rate at anaerobic threshold, peak heart rate, anaerobic threshold RER, peak RER, minute Ventilation (VE), VE/VCO_2_ slope. and ΔVO_2_/ΔWR (Table 2). Of the different exercise-based CR strategies, the overall improving effects were as follows: IVR > ITM > COR > IVA > COA. However, for data collected from the second recording point (three months after the discharge). The difference regarding the improving effect between the Control group and CR groups was reduced (Table 3), and even the difference between different CR strategies was reduced (Table 3). The overall improving effects three months after discharge were as follows: IVR > ITM > COR > IVA > COA.

**Table 2:** Effects of different exercise-based CR on the cardio-pulmonary function of AMI patients receiving PCI one month after discharge.

Indicator	Control (n = 21)	COR (n = 18)	COA (n = 19)	IVA (n = 21)	IVR (n = 22)	ITM (n = 26)	p-value
Heart rate at anaerobic threshold (BPM)	106 ± 17	105 ± 16	103 ± 12	101 ± 10	106 ± 12	104 ± 10	0.53
Peak heart rate (BPM)	121 ± 12	119 ± 11	122 ± 15	120 ± 13	123 ± 11	122 ± 12	0.95
Oxygen uptake at anaerobic threshold (mL/min)	0.71 ± 0.11	0.78 ± 0.08	0.72 ± 0.10	0.74 ± 0.12	0.81 ± 0.09	0.79 ± 0.11	**0.03**
Peak oxygen uptake (mL/min)	0.95 ± 0.27	1.13 ± 0.21	1.07 ± 0.18	1.02 ± 0.21	1.31 ± 0.11	1.29 ± 0.14	**0.00**
Metabolic equivalent at anaerobic threshold (mets)	2.9 ± 0.4	3.2 ± 0.4	3.1 ± 0.3	3.1 ± 0.2	3.7 ± 0.6	3.4 ± 0.3	**0.00**
Peak metabolic equivalent (mets)	3.87 ± 0.47	4.22 ± 0.52	4.11 ± 0.57	3.92 ± 0.48	4.43 ± 0.51	4.05 ± 0.41	**0.00**
Maximum oxygen pulse (ml/Beats)	7.61 ± 1.65	8.24 ± 1.86	7.82 ± 2.02	8.03 ± 1.31	8.85 ± 1.42	8.45 ± 1.52	**0.00**
Anaerobic threshold RER	0.99 ± 0.11	0.98 ± 0.09	1.02 ± 0.08	1.05 ± 0.10	0.94 ± 0.11	0.96 ± 0.12	0.10
Peak RER	1.15 ± 0.15	1.14 ± 0.12	1.14 ± 0.16	1.11 ± 0.12	1.14 ± 0.14	1.13 ± 0.11	0.72
VE (L/min)	24.2 ± 3.1	23.1 ± 3.8	25.2 ± 4.4	24.1 ± 3.8	25.9 ± 4.5	25.1 ± 5.2	0.20
VE/VCO_2_ slope	31.9 ± 6.2	30.8 ± 5.2	29.6 ± 4.4	30.2 ± 4.1	28.1 ± 5.8	29.2 ± 5.2	0.06
ΔVO_2_/ΔWR (mL/min/W)	8.8 ± 2.6	9.4 ± 1.8	9.1 ± 2.5	8.9 ± 2.3	9.5 ± 1.7	9.4 ± 2.0	0.66

**Table 3:** Effects of different exercise-based CR on the cardio-pulmonary function of AMI patients receiving PCI three month after discharge.

Indicator	Control (n = 21)	COR (n = 18)	COA (n = 19)	IVA (n = 21)	IVR (n = 22)	ITM (n = 26)	p-value
Heart rate at anaerobic threshold (BPM)	112 ± 13	115 ± 11	116 ± 14	111 ± 10	109 ± 13	114 ± 14	0.75
Peak heart rate (BPM)	128 ± 13	129 ± 14	132 ± 12	130 ± 11	132 ± 14	127 ± 12	0.12
Oxygen uptake at anaerobic threshold (mL/min)	0.86 ± 0.14	0.88 ± 0.11	0.82 ± 0.10	0.84 ± 0.12	0.87 ± 0.19	0.89 ± 0.16	0.38
Peak oxygen uptake (mL/min)	1.22 ± 0.30	1.23 ± 0.26	1.18 ± 0.21	1.22 ± 0.16	1.36 ± 0.15	1.30 ± 0.12	0.09
Metabolic equivalent at anaerobic threshold (mets)	3.4 ± 0.4	3.3 ± 0.4	3.2 ± 0.4	3.0 ± 0.3	3.7 ± 0.7	3.3 ± 0.4	0.00
Peak metabolic equivalent (mets)	4.81 ± 0.57	5.32 ± 0.62	5.12 ± 0.51	4.92 ± 0.52	5.43 ± 0.61	5.05 ± 0.54	0.00
Maximum oxygen pulse (mL/Beats)	9.11 ± 2.45	9.44 ± 2.86	9.82 ± 3.02	9.43 ± 1.31	10.05 ± 2.82	10.45 ± 1.62	0.18
Anaerobic threshold RER	1.01 ± 0.12	0.99 ± 0.09	0.97 ± 0.07	1.02 ± 0.10	1.04 ± 0.12	0.97 ± 0.10	0.10
Peak RER	1.17 ± 0.17	1.16 ± 0.11	1.18 ± 0.12	1.09 ± 0.14	1.17 ± 0.13	1.14 ± 0.11	0.06
VE (L/min)	39.2 ± 4.3	40.1 ± 6.2	37.2 ± 5.2	38.1 ± 4.3	40.9 ± 6.5	39.1 ± 6.2	0.15
VE/VCO_2_ slope	30.9 ± 5.2	30.8 ± 5.2	28.1 ± 5.4	29.2 ± 3.4	28.3 ± 5.2	28.9 ± 4.2	0.7
ΔVO_2_/ΔWR (mL/min/W)	9.8 ± 1.9	9.8 ± 1.1	10.1 ± 2.2	9.9 ± 1.8	10.5 ± 1.5	9.8 ± 1.3	0.77

### Effects of different exercise-based CR strategies on the hemodynamics parameters of AMI patients receiving PCI

The data regarding end-diastolic anteroposterior diameter and Left Ventricular Ejection Fraction (LVEF) were also collected from records at the time of admission and three months after discharge. The analysis results showed that at the admission, no significant difference was detected between different groups (Table 4). However, after the three-month follow-up, the patients in all the exercise-based CR strategies showed improving effect on the LVEF compared with the patients in the Control group (Table 5) (p < 0.05). The improved effects were not observed for end-diastolic anteroposterior diameter (Table 5). Of different exercise-based CR strategies, the overall improving effects three months after discharge were as follows: IVR > COR > ITM > COA > IVA.

**Table 4:** Effects of different exercise-based CR on the cardio-pulmonary function of AMI patients receiving PCI upon admission.

Indicator	Control (n = 21)	COR (n = 18)	COA (n = 19)	IVA (n = 21)	IVR (n = 22)	ITM (n = 26)	p-value
LVEDD (mm)	47.9 ± 7.0	48.1 ± 8.8	47.2 ± 7.5	46.4 ± 8.2	46.2 ± 7.6	48.2 ± 7.8	0.08
LVEF (%)	55.0 ± 4.8	57.3 ± 6.1	55.1 ± 5.2	56.9 ± 7.8	57.0 ± 7.5	55.8 ± 6.3	0.40

**Table 5:** Effects of different exercise-based CR on the cardio-pulmonary function of AMI patients receiving PCI three month after discharge.

Indicator	Control (n = 21)	COR (n = 18)	COA (n = 19)	IVA (n = 21)	IVR (n = 22)	ITM (n = 26)	p-value
LVEDD (mm)	48.6 ± 6.1	47.3 ± 6.8	46.1 ± 5.5	47.2 ± 7.2	44.1 ± 6.2	43.4 ± 4.8	0.02
LVEF (%)	57.5 ± 6.0	61.4 ± 7.1	57.2 ± 4.2	59.7 ± 5.7	62.5 ± 7.3	60.5 ± 6.2	0.04

### Effects of different exercise-based CR strategies on the 6MWT and life quality of AMI patients receiving PCI

The walking distance assessed with the 6MWT test did not differ between the groups before the beginning of rehabilitation (Table 6). After the three-month rehabilitation, an increase in the walking distance was noted in all the CR groups compared to the Control group (Table 7) (p < 0.05), and the overall improving effects were as follows: IVR > ITM > COR > IVA > COA. Regarding the patient's quality of life assessed with the WHOQOL-BREF questionnaire, data did not differ between the groups at the beginning of rehabilitation, but similar to the changing pattern of 6MWT, improvement in the patient's quality of life was observed in all CR groups compared to Control group after three months (Table 6) (p < 0.05), and the overall improving effects were as following: IVR > ITM > COR > IVA > COA (Table 7).

**Table 6:** Effects of different exercise-based CR on the cardio-pulmonary function of AMI patients receiving PCI upon admission.

Indicator	Control (n = 21)	COR (n = 18)	COA (n = 19)	IVA (n = 21)	IVR (n = 22)	ITM (n = 26)	p-value
6MWT (m)	341.6 ± 62.9	344.1 ± 68.8	337.2 ± 66.6	346.4 ± 58.4	348.9 ± 63.7	338.2 ± 58.3	0.30
Life quality	3.29 ± 0.47	3.35 ± 0.61	3.55 ± 0.52	3.49 ± 0.78	3.52 ± 0.51	3.38 ± 0.63	0.14

**Table 7:** Effects of different exercise-based CR on the cardio-pulmonary function of AMI patients receiving PCI three month after discharge.

Indicator	Control (n = 21)	COR (n = 18)	COA (n = 19)	IVA (n = 21)	IVR (n = 22)	ITM (n = 26)	p-value
6MWT (m)	378.6 ± 71.4	432.2 ± 62.3	456.3 ± 57.6	488.6 ± 66.6	496.3 ± 58.1	474.9 ± 62.6	0.00
Life quality	3.57 ± 0.55	3.92 ± 0.66	3.77 ± 0.71	3.86 ± 0.65	4.22 ± 0.67	4.09 ± 0.68	0.00

## Discussion

Revascularization is the currently most widely employed treatment strategy recommended for CHD patients with severe stenosis arteries.^19^ As a typical type of CHD, the prognosis of patients who have undergone PCI is obviously better than seemingly similar patients who have not, which is attributed to the quick restoration of blood circulation of coronary arteries in patients with AMI.^20^ In recent years, the incidence and mortality of myocardial (AMI) have increased, and thus PCI has been universally applied to AMI patients as the first treatment option. However, the effects of PCI are rendered less effective by them leading to further cardiomyocyte death known as Ischemia-Reperfusion (I/R) injury. Contrary to the rapid development of PCI technology, few means for handling complications associated with PCI are available. Therefore, the prevention of the recurrence of AMI after PCI and secondary injuries such as I/R injury after PCI is key to the prognosis of AMI patients receiving PCI.

CR is a new concept of heart protection proposed in recent years, which is to promote the physical, psychological, and social functions of CHD patients via the use of a variety of intervention methods. Exercise rehabilitation is the core part of CR, which can improve cardiopulmonary exercise capacity,^21^ oxygen uptake efficiency slope,^22^ flow-mediated vasodilation,^23^ while can reduce inflammation,^24^ the incidence of coronary restenosis,^25^ and the overall mortality and cardiac mortality in AMI patients receiving PCI.^15^ Based on the study by Ghashghaei et al., exercise-based CR strategies before and after surgery could significantly reduce the psychological burden of patients with CHD, improving the psychological mood, enthusiasm for medical treatment, and heart function of the patients.^26^^,^^27^ The other study by Ji et al. showed that for elderly CHD patients receiving PCI surgery, exercise-based CR strategies could effectively improve patients' exercise tolerance, reduce the incidence of myocardial ischemia, and improve their quality of life.^28^ However, the current exercise-based CR strategies lack standard protocols, and different institutes employ different training strategies. The lack of comparison between different exercises has influenced the further application of exercise-based CR strategies in the clinic. Thus, in the current analysis, the authors performed a comparison of the improving effects of different exercise types on the prognosis of AMI patients receiving PCI surgery.

The data showed that different exercise types all improved the prognosis of patients compared with normal nursing strategy after the first month of discharge from the hospital. Of these exercises, IVR showed much stronger improving effects even assessed by different criteria such as cardio-pulmonary function, hemodynamics parameters, 6MWT, and life quality. Nevertheless, at the second recording point (three months after the discharge), the difference regarding the improving effects between exercise-based CR strategies and normal nursing strategy or between different exercise-based CR strategies reduced dramatically, which might indicate that the timely exercise training achieved better-improving effects, and the effects would decline with time. Compared with other exercise types, IVR showed obviously better-improving effects. Previous studies showed that high-intensity IVR exercises would improve oxygen absorption as well as distribution into skeletal muscles, and improve vascular function.^29^ The results were further verified in elderly CHD patients, and the data also confirmed the safety of the application of IVR to patients of different ages.^30^

Collectively, by comparing the prognosis of AMI patients receiving PCI subjected to different exercise-based CR strategies, the current study showed that exercise-based CR strategies had better improving effects than normal nursing strategy on the prognosis of AMI patients after PCI surgery, but the improving effects would decline with time. Thus, the earlier the patients accept exercise-based CR strategies, the better outcome will be achieved. Moreover, of the different exercise types, IVR exercise showed much better effects than other strategies regardless of assessing criteria. However, the current study only provided a preliminary conclusion on the effect of different exercise-based CR strategies in that the current study was a retrospective analysis with a small sample size in a single center. To verify the present conclusion, more comprehensive clinical trials with larger sample sizes and multiple centers are needed in the future.

## Authors' contributions

Huiying Liang: Performed conceptualization, data curation, formal analysis, and writing-original draft.

Xinhua Hu: Performed conceptualization, data curation.

Hongying Liao: Performed conceptualization and writing review & editing.

## Funding

Not applicable.

## Conflicts of interest

The authors declare no conflicts of interest.
